# Adult Popillia japonica as an Otorhinolaryngologic Invasive Foreign Body in a Rural Area

**DOI:** 10.7759/cureus.12046

**Published:** 2020-12-12

**Authors:** Lindsey Schwanke, Derek Chen, Christine M Lomiguen, Justin Chin

**Affiliations:** 1 Primary Care, Lake Erie College of Osteopathic Medicine, Erie, USA; 2 Primary Care, Lake Erie College of Osteopathic Medicine, Greensburg, USA; 3 Pathology, Lake Erie College of Osteopathic Medicine, Erie, USA; 4 Medical Education, Lake Erie College of Osteopathic Medicine, Erie, USA; 5 Family Medicine, LifeLong Medical Care, Richmond, USA

**Keywords:** bug, irrigation, japanese beetle, foreign body retrieval, insect, external ear, popillia japonica, beetle, external auditory canal, hearing loss

## Abstract

Otorhinolaryngologic foreign bodies may be encountered in-office visits, the emergency department, and speciality consultations. These include food, toys, and other small items, are present in pediatric patients. Because patients may be asymptomatic and the insertion of the foreign body not observed, obtaining medical care may be delayed. Conversely, insects as foreign bodies, especially in the external ear canal, can cause a patient significant pain and distress, directing the patient to seek immediate care. Here, we present a case of an adult Japanese beetle (*Popillia japonica*) as a foreign body in the ear of a 14-year-old female. A review of otorhinolaryngologic foreign bodies is also discussed, with particular attention to the ear and rural location. This case highlights the potential for agricultural insects to act as invasive foreign bodies, especially in areas where they are known to be endemic pests and the consequences of delayed treatment.

## Introduction

Popillia japonica, more commonly known as the Japanese beetle, was discovered in the United States in the early 1900s [1]. Originally native to Japan, the lack of natural predators has allowed for its proliferation and spread. The Japanese beetle is most predominantly found in the Midwest and East Coast of the United States, with only nine states considered pest-free [2,3]. The life cycle of the beetle brings about agricultural destruction, as its various stages result in the skeletonization of foliage and destruction of fruits and roots. Japanese beetles reach maturity toward the end of spring and middle of summer. A green head and body recognize them with copper, brown-coloured wings, approximately 10mm in length [4]. Studies on the Japanese beetle are primarily focused on containment and eradication strategies to decrease spread and damage to crops [5,6]. Limited research exists on medical complications related to the Japanese beetle as it is not known to harm humans or livestock directly.

Similar to many other insects that burrow into the ground for safety, protection, or reproduction, the Japanese beetle can be found invading human orifices. A typical location is the external auditory canal (EAC) due to its dark and warm environment. Compared to other foreign bodies, live insects account for 14-18% of all cases found in the EAC [7]. Ranging from asymptomatic irritation to pain and possible hearing loss, a patient presentation can vary depending on the type and size of an insect, location in the EAC, and duration of exposure [8,9]. Direct visualization and prompt removal are critical in relieving the physical and emotional discomforts of the foreign body as well as preventing future sequelae [10]. This case report focuses on the presence of a foreign insect body, specifically the Japanese beetle, in the ear canal of a 14-year-old female and reviews treatment considerations and challenges in rural settings.

## Case presentation

Ms S is a 14-year-old Caucasian female who presented to the emergency department with her mother after a one-hour history of acute otalgia and crawling sensation in the right ear. The pain started while swimming in a local pool, in a rural Pennsylvania township at night; the pain increased as time progressed. A friend’s mother attempted to use a bulb syringe to suction any trapped water or foreign bodies. However, the patient was unable to notice any difference. The patient denied any otorrhea, hearing loss, tinnitus, or vertigo.

**Figure 1:**
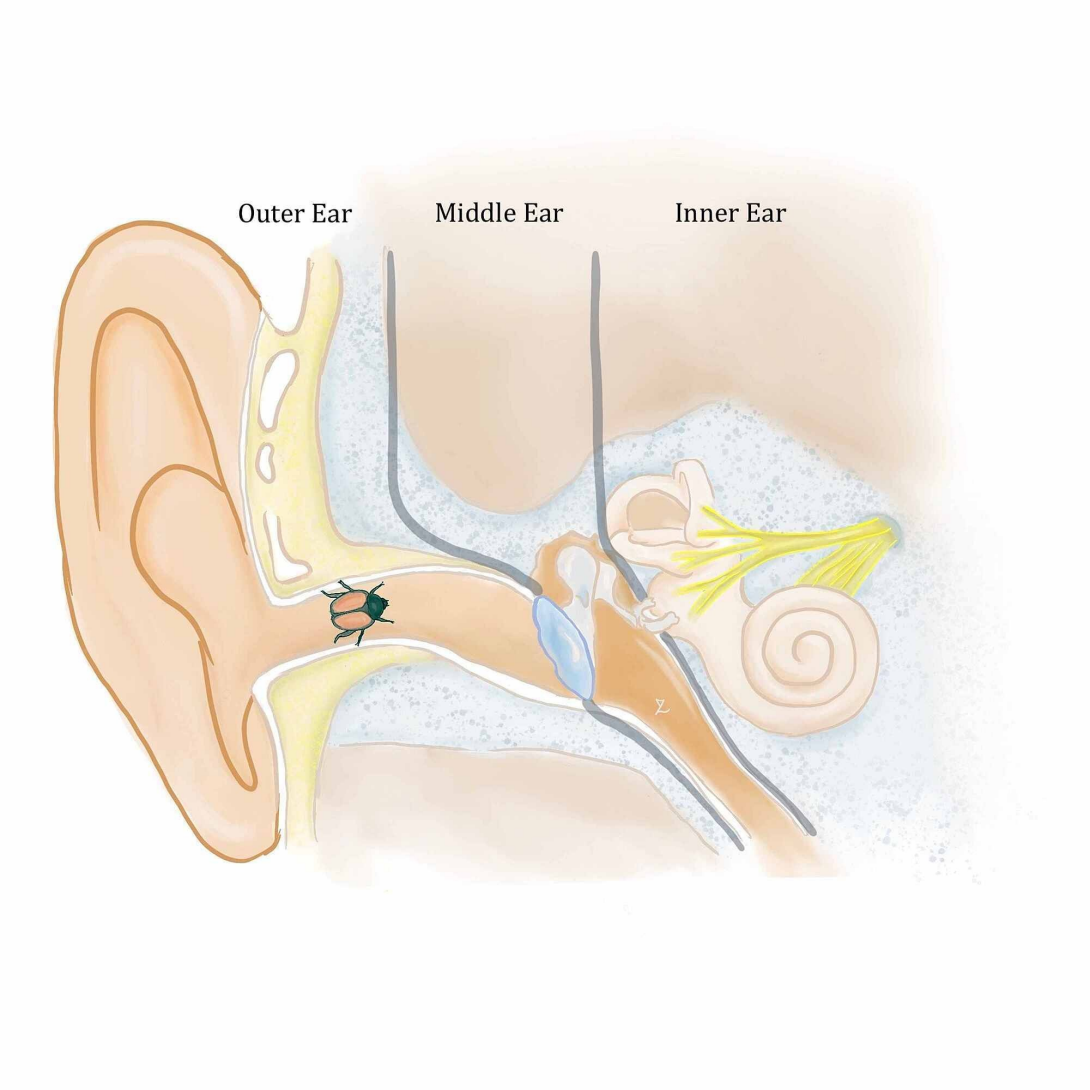
Diagram of the ear with approximate location of the Japanese beetle. (Original illustration created by YaQun Zhou)

Vitals were within normal limits. Physical exam revealed a normal left ear, with clear EAC and mobile tympanic membrane on otoscopic examination. In the right ear, a large, black insect was visualized 1.5cm deep into the EAC, consistent with an insect (Figure 1). The right EAC appeared erythematous, and the tympanic membrane was not able to be seen. Initially believed to be dead, two attempts were made using a standard ear lavage to irrigate the EAC. However, the patient noted increased pain. Upon revisualization, movement of the insect was seen. The patient declined a third attempt at irrigation, and mineral oil was instilled to kill the insect. The patient was discharged with over the counter pain medication and advised to follow-up with an outpatient otolaryngologist to remove the insect.

On the following day, he dead beetle was removed with alligator forceps and identified to match an adult Japanese beetle physiologically. The otoscopic exam revealed an erythematous right EAC with a regular, non-perforated tympanic membrane. Initial audiological evaluation revealed mild conductive hearing loss; however, a repeat audiogram one week following the incident was normal.

## Discussion

The external auditory canal (EAC) is separated into a cartilaginous (lateral) section and a bony section (medial), with narrowing of the canal at this transition. The tympanic membrane serves as a divider between the external and middle ear zones. The bony section of the EAC is more receptive to pain due to lack of subcutaneous tissue between the skin and periosteum, resulting in a higher risk of trauma upon instrumentation [11]. With aural foreign bodies, symptom presentation can be dependent on the ultimate site of deposition in the EAC as deeper objects are more likely to have more pronounced symptoms [12]. For pediatric populations, small objects such as beads, plastic toys, pebbles, or popcorn kernels are commonly seen in primary care settings. They can often be manipulated and removed without specialist intervention [13]. Mobile objects such as insects or other arthropods present an increased challenge as insects can cause further damage when attempting to escape retrieval efforts by burrowing through the tympanic membrane and entering the middle ear space [14]. The psychological effect of knowing that a living organism is trying to enter the body can further exacerbate perceptions of pain and increase anxiety for while waiting for treatment.

Geographic location can play a role in the type of insect or arthropod found in the EAC. In rural surroundings, ticks and mites are commonly found on livestock and can subsequently result in human otocariasis or infestation of the human EAC [15]. In comparison, cockroaches and other flying insects have been seen in the EAC of inner-city youth and the homeless community [16]. Historically, identification of foreign insect bodies was reliant on local knowledge of endemic pests, however with globalization and international travel, invasive species are being discovered in new areas [17]. As seen in this case, the Japanese beetle is not an endemic insect for the area, which highlights the need for physicians to consider atypical possibilities in foreign insect bodies. Successful removal relies on immobilization and neutralization of live insects. Irrigation with ethanol, lidocaine, or viscous liquids such as mineral oil, have comparable efficacy in killing the insect and allowing for removal under direct visualization [18,19]. In settings where irrigation may not be possible, keeping the insect alive and using light-assisted removal via otoscope can also be a viable option [20]. Regardless of the method, physicians need to choose a modality that they are proficient in as increased first-attempt retrieval has been shown to decrease potential complications associated with foreign body removal.

## Conclusions

Insects in the ear canal can cause damage that results in both acute and lasting effects. Physicians need to recognize insects as possible etiologies, especially in rural locales, where specialist care may not be readily available. As seen with the case above of the Japanese beetle, direct visualization and prompt irrigation have been shown to mitigate potential sequelae and decrease patient discomfort. Future studies can further categorize and optimize foreign insect bodies in the ears.
